# Supplementary material for: Temozolomide and Lomustine Induce Tissue Factor Expression and Procoagulant Activity in Glioblastoma Cells In Vitro
Source: Cancers (Basel). 2023 Apr 18;15(8):2347. doi: 10.3390/cancers15082347 (PMC10137012; doi:10.3390/cancers15082347)
Supplement: Supplementary file 1 [file cancers-15-02347-s001.zip › Table_S1.pdf]

Table S1. Primer sequences used for qPCR.

Gene	Species	Forward primer	Reverse primer
F3	human	TACAGACAGCCCGGTAGAGT	AGCTCCAACAGTGCTTCCTT
IL1B	human	ATGATGGCTTATTACAGTGGCAA	GTCGGAGATTCGTAGCTGGA
IL6	human	GCAGAAAAAGGCAAAGAATC	CTACATTTGCCGAAGAGC
CXCL8	human	AGGTGCAGTTTTGCCAAGGA	TTTCTGTGTTGGCGCAGTGT
TNFA	human	CCTCTCTCTAATCAGCCCTCTG	GAGGACCTGGGAGTAGATGAG
GAPDH	human	TTCCAGGAGCGAGATCCCT	CACCCATGACGAACATGGG
ACTB	human	GATCAAGATCATTGCTCCTC	TTGTCAAGAAAGGGTGTAAC
