# Supplementary figures and images for: Temozolomide and Lomustine Induce Tissue Factor Expression and Procoagulant Activity in Glioblastoma Cells In Vitro
Source: Cancers (Basel). 2023 Apr 18;15(8):2347. doi: 10.3390/cancers15082347 (PMC10137012; doi:10.3390/cancers15082347)

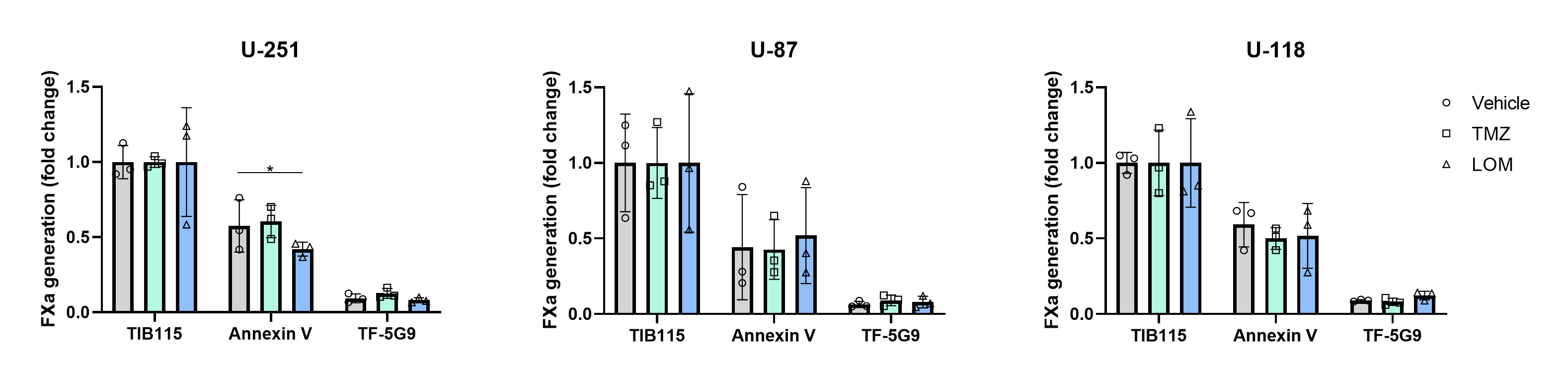

Supplement: Supplementary file 1 [file cancers-15-02347-s001.zip › Figure_S2.tif]
